# Factors associated with low birthweight among newborns delivered at public health facilities of Nekemte town, West Ethiopia: a case control study

**DOI:** 10.1186/s12884-019-2372-x

**Published:** 2019-07-02

**Authors:** Shimelis Girma, Teshale Fikadu, Eskeziyaw Agdew, Desta Haftu, Genet Gedamu, Zeritu Dewana, Bereket Getachew

**Affiliations:** 1 Department of Psychiatry, Institute of Health, Jimma University, Jimma, Ethiopia; 2 Department of Public Health, College of Medicine and Health Science, Arba Minch University, Arba Minch, Ethiopia; 3 Department of Midwifery, Arba Minch College of Health Sciences, Arba Minch, Ethiopia; 4 Department of Statistics, College of Natural Science, Arba Minch University, Arba Minch, Ethiopia

**Keywords:** Low birthweight, Maternal nutritional status, Nekemte town

## Abstract

**Background:**

Low birthweight (LBW) remains the most important risk factor which attributed to mortality of 15–20% of newborns across the globe. An infant with low birthweight is more likely to have stunting in childhood and develop markers of metabolic risk factors at his later age. Furthermore, LBW is a risk for inter-generational assaults of malnutrition as it is the risk for sub optimal growth until adulthood, affecting women’s and male’s reproductive capabilities. Thus, there is enough concern to study the determinants of LBW across different settings. Accordingly, this study was conducted to assess the determinants of low birthweight s in public health facilities of Nekemte town, West Ethiopia.

**Methods:**

Facility based unmatched case control study was employed from February to April 2017. The data were collected using structured, pretested interviewer administered questionnaire in all public health facilities of Nekemte town. Consecutive live births of less than 2500 g in each of the hospitals and health centres were selected as cases and succeeding babies with weights of at least 2500 g as controls. Data were entered in to Epi-data software version 3.1 and exported to SPSS Version 21 and analyzed using frequency, cross-tabs and percentage. Factors with *p*-value < 0.25 in Bivariate analysis were entered in to multivariable logistic regression and statistical significance was considered at *p*-value < 0.05.

**Result:**

A total 279 (93 cases &186 controls) were included in the study with a mean birthweight of 2138.3 g ± SD 206.87 for cases and 3145.95 g ± SD 415.98 for controls. No iron-folate supplementation (AOR = 2.84, 95% CI, 1.15–7.03), no nutritional counselling (AOR = 4.05, 95%CI, 1.95–8.38), not taking snacks (AOR =3.25, 95%CI, 1.64–6.44), maternal under nutrition (AOR =5.62, 95%CI, 2.64–11.97), anemia (AOR = 3.54, 95%CI, 1.46–8.61) and inadequate minimum dietary diversity score of women MDDS-W (AOR = 6.65, 95%CI, 2.31–19.16) were factors associated with low birthweight .

**Conclusion:**

Lacking nutrition counselling during pregnancy, lacking iron/folic acid supplementation during pregnancy, not taking snacks during pregnancy, maternal under-nutrition, maternal anemia and inadequate minimum dietary diversity score of women (MDDS-W) were independently associated with LBW. Thus, public health intervention in the field of maternal and child health should address these determinants.

## Background

The World Health Organization (WHO) defined low birthweight (LBW) as weight less than 2500 g at birth. Low birthweight contributes to a variety of pitiable health outcomes [1].

The majority of LBW in low income countries is due to IUGR, while it is mostly due to preterm birth in high income countries. Although in many cases, the causes of prematurity are vague, they may include maternal high blood pressure, acute infections, hard physical work, multiple births, stress, anxiety, and other psychological factors such as gender-based violence. The causes of IUGR include, poor nutritional status of the mother at conception, low weight gain during pregnancy due to insufficient dietary intake or extra expenditure of calories (hard work), short maternal height due to youthful under-nutrition and infections, anemia, acute and chronic infections that could result in under-nutrition and consecutive poor pregnancy outcomes including LBW [2].

LBW is a global public health challenging problem. Its high priority stems from the fact that it is the major determinant of infant morbidity and that it contributes markedly to the overall burden of childhood death. LBW has also been linked to the high prevalence of stunting seen in low income countries and may be important in the ethology of chronic dietary diseases such as obesity, diabetes and cardiovascular diseases in adulthood [2].

Worldwide more than 20 million low birthweight occur annually with the incidence of 15 to 20%, majority of this occur in low- and middle-income countries and 95.6% occur in developing nations. Its regional estimate was 28% in South Asia, 13% in sub-Saharan Africa and 13% in least developed country [1, 3] As EDHS 2011 report in Addis Ababa, Ethiopia 11.4% are LBW [4].

Being born with LBW is generally recognized as a disadvantage for the infant. Among all neonatal death 60 to 80% occur due to LBW. It is an important cause of perinatal mortality and both short- and long-term infant and childhood morbidity. Mortality rate of LBW infant were up to 40 times higher than infants with birthweights of at least 2500 g, and they are many times more likely to end up with long-term handicapping conditions [5–11].

A recent study done in India has reported that maternal age (< 19 years), rural residence, maternal weight (< 45 kg), gestational age (< 37 weeks), bad obstetric history and Pregnancy-induced hypertension have a strong association with low birthweight. A number of studies have shown correlates of infant’s maternal nutritional status, young maternal age, bad obstetric history, maternal anemia and rural settlements, antenatal care received, prematurity, the birth interval with low birthweight [12–29]. However, the majority of these studies did not address maternal nutritional status and maternal dietary practices. Therefore, the aim of this study was to determine nutritional and others factors associated with LBW among newborns delivered at public health facilities of Nekemte town, West Ethiopia.

## Methods and materials

### Study setting and sampling

An institution-based unmatched case control study was employed in public health facilities of Nekemte town from March to April, 2017. The town is located 340 km west of Addis Ababa (the capital city of Ethiopia). It had an estimated total population of 110, 688 people in the year 2016/17 which is projected from the 2007 Ethiopia Central Statistical Agency. There are two public health centres and two hospitals providing delivery service for pregnant women and these public health facilities were included in the study.

Cases were all live births of less than 2500 g while controls were all live birthweight of at least 2500 g.

Sample size was calculated using Epi info version 7 by assuming the proportion of women with anemia among controls and cases were 11.6 and 25.8% respectively, 95% CI, 80% power, case to control ratio of 1:2 and accounted for 10% possible non-response. The total sample size was 279 (93 cases and 186 controls). To calculate sample size, maternal anemia was chosen as an independent variable since it gave maximum sample size. The two hospitals and two health centres found in Nekemte town were included. The sample is allocated proportionally based on the number of births in a 5 months period. Consecutive sampling was used to select cases and systematic random sampling was used to select controls. Controls were selected from the same health facility from which cases were selected.

### Data collection and measurements

Semi structured interviewer administered questionnaires that contain; nutritional and dietary related factors, socio demographic and socio-economic related factors, medical and obstetrics related factors, behaviour related factors, environmental related factors and infant related factors. The interviews were conducted at the health facility after the mother had given birth. Questionnaires were prepared in English and translated to local language (Afaan Oromo) by language experts and retranslated back to English by another person to check consistency. Food security status of households was determined based on nine standard household food insecurity (HFIAS) questions that were validated for a low income country. Maternal dietary diversity (MDD-W) was determined by using 24-h dietary recall method by MDD-W. Maternal haemoglobin level was reviewed from client card from each public health facilities to determine anaemia.

The neonate’s weight was measured using a balanced Seca scale (Germany) to the nearest 0.01 g within 1 hours of birth. The mid-upper arm circumference (MUAC) of the mother was measured right after delivery using flexible non-stretchable standard tape measure.

### Data analysis

Data was checked for completeness, coded and entered into Epi data version 3.1 and exported to SPSS version 21.0 statistical software for analysis. Descriptive statistics were presented using standard statistical parameters such as percentages, means and standard deviations. Bivariate analysis was done and all explanatory variables with a *p*-value less than 0.25 were included in multivariable analysis. Multi variable conditional logistic regression analysis was employed to determine independent determinant factors.

Wealth index was calculated as a composite indicator of living standard by considering variables related to ownership of household assets. The computation was prepared using principal component analysis and a continuous variable was generated by summing up the principal components into one. Household food insecurity access scale scores (0–27), were summed to produce an index of household food insecurity. As to maternal dietary diversity scores, a pregnant woman was assigned to be adequate if her MDD-W score was > 5 or inadequate with a MDD-W score < 5. Hosmer and lemeshow test was used to asses model fitness (*p* = 0.75) and variance inflation factor (VIF) and tolerance test were used to check multicollinearity.

## Results

Total of 279 (93 cases and 186 controls) were participants in the study. Mean birthweight of cases and controls were 2138.28 g ± 206.87 and 3145.16 g ± 414.99. Majority of the newborns were males 49 (52.7%) for cases and 117(62.9%) for controls. Fifty-two (55.9%) of cases and 111(59.7) of controls came from an urban setting. Mothers of 61cases (65.6%) and 154 controls (82.5%) did not attend formal education. (Table 1).

**Table 1:** Socio-economic and demographic characteristics of mothers of the study participants in Nekemte town, West Ethiopia, 2017

Variables	Case No (%)	Controls No (%)	*p*-value
Infant sex			
Male	49 (52.7)	117 (62.9)	
Female	44 (47.3)	69 (37.1)	0.052
Maternal age (year)			
≤ 20	9 (9.7)	32 (17.2)	0.001
21–35	77 (82.8)	147 (79.6)	
> 35	7 (7.5)	6 (3.2)	
Residence			
Rural	41 (44.1)	75 (40.3)	0.28
Urban	52 (55.9)	111 (59.7)	
Religion			
Muslim	69 (74.2)	140 (75.3)	0.68
Orthodox	20 (21.5)	42 (22.6)	
Others	4 (4.3)	4 (2.2)	
Ethnicity			
Amhara	77 (82.8)	168 (90.3)	0.27
Oromo	10 (10.8)	7 (3.8)	
Others	6 (6.5)	11 (5.9)	
Marital status			
Married	87 (93.5)	172 (92.5)	0.38
Single/ divorced	6 (6.5)	14 (7.5)	
Educational status of mothers			
Informal education	32 (34.4)	32 (17.2)	0.001
Formal education	61 (65.6)	154 (82.8)	
Occupation of mothers			
Employed	17 (18.3)	63 (33.9)	0.001
Merchant	5 (5.4)	24 (12.9)	
Housewife	65 (69.9)	91 (48.9)	
Others	6 (6.5)	8 (4.3)	
Wealth index			
Lower	58 (62.4)	101 (54.3)	0.53
Middle	16 (17.2)	49 (26.3)	
Upper	19 (20.4)	36 (19.4)	

Mean maternal height for cases and controls was 155 cm ± 0.07 and 159 cm ± 0.07. Under-nutrition in mothers as defined by MUAC < 23 cm was 52.7% for cases and 13.4% for controls (Table 2).

**Table 2:** Nutritional related characteristics of mothers of study participants in Nekemte town, West Ethiopia, 2017

Variables	Case No (%)	Controls No (%)	*p*-value
MUAC			
< 23	49 (52.7)	25 (13.4)	0.001
> 23	44 (47.3)	161 (86.6)	
Iron and folate supplementation			
Yes	64 (68.8)	166 (89.2)	0.001
No	29 (31.2)	20 (10.8)	
Any multivitamin			
Yes	9 (9.7)	36 (19.4)	0.018
No	84 (90.3)	150 (80.6)	
Nutritional counseling during pregnancy			
Yes	40 (43.0)	153 (82.3)	0.001
No	53 (57.0)	33 (17.7)	
Taking snacks during pregnancy			
Yes	34 (36.6)	134 (72)	0.001
No	59 (63.4)	52 [27]	
Minimum dietary diversity score of women			
Inadequate	87 (93.5)	117 (62.9)	0.001
Adequate	6 (6.5)	69 (37.1)	
Anemia			
Yes	30 (32.3)	17 (9.1)	0.001
No	63 (67.7)	169 (90.9)	
Eating out of home			
Yes	81 (87.1)	167 (89.8)	0.251
No	12 (12.9)	19 (10.2)	
Chat chewing			
Yes	33 (35.5)	50 (26.9)	0.071
No	60 (64.5)	136 (73.1)	
Alcohol consumption			
Yes	5 (5.4)	15 (8.1)	0.215
No	88 (94.6)	171 (91.9)	

Findings of multivariable logistic regression indicated that lack of maternal iron and folic acid supplementation during pregnancy (AOR = 2.84, 95%CI, 1.15–7.03), lack of nutritional counselling during the current pregnancy (AOR = 4.05, 95% CI, 1.95–8.38), not taking snacks (AOR = 3.25, 95%CI, 1.64–6.44), maternal under nutrition (AOR = 5.62, 95%CI, 2.64–11.97), and maternal anaemia (AOR = 3.54, 95%CI, 1.46–8.61) were positively associated with low birthweight (Table 3).

**Table 3:** Factors independently associated with LBW among newborns delivered in public health facilities of Nekemte town, West Ethiopia, 2017

Variable	Case No (%)	Controls No (%)	COR (95%CI)	AOR (95% CI)
Iron and foliate supplementation				
Yes	64 (68.8)	166 (89.2)	1	1
No	29 (31.2)	20 (10.8)	3.76 (1.99,7.12)	2.84 (1.15,7.03)
Nutritional counseling				
Yes	40 (43.0)	153 (82.3)	1	1
No	53 (57.0)	33 (17.7)	6.14 (3.52,10.72)	4.05 (1.95, 8.38)
Taking snacks during pregnancy				
Yes	34 (36.6)	134 (72)	1	1
No	59 (63.4)	52 [27]	4.47 (2.63, 7.61)	3.25 (1.64, 6.44)
Maternal MUAC				
< 23	49 (52.7)	25 (13.4)	7.17 (3.99,12.88)	5.62 (2.64,11.97)
≥ 23	44 (47.3)	161 (86.6)	1	1
Anemia				
Yes	30 (32.3)	17 (9.1)	4.73 (2.44, 9.17)	3.54 (1.46,8.61)
No	63 (67.7)	169 (90.9)	1	1
Minimum dietary diversity score of women				
Inadequate	87 (93.5)	117 (62.9)	8.55 (3.55,	6.65 (2.31,19.16)
Adequate	6 (6.5)	69 (37.1)	20.61)1	1

## Discussion

LBW is a global challenging public health problem. Its high priority stems from the fact that it is the major predictor of infant morbidity and mortality [30].

In this study mothers not counselled about nutrition during pregnancy had significantly higher odds of LBW than their counterparts. Nutritional counselling may improve their feeding behaviour and hence, their nutritional status which may help mothers to decrease the risk of delivering LBW babies. The finding was consistent with studies done in Ethiopia [20, 31]. The risk of low birthweight was higher among mothers who did not take snacks during the current pregnancy, consistent with studies done in Ethiopia and Ghana [13, 21]. Mothers who were counselled about feeding practices during pregnancy were 88% less likely to give birth to LBW infants than their counterparts. There is mounting evidence from controlled trials that improving food intake during pregnancy effectively reduces the risk of giving birth to LBW babies [26, 27]. Similarly, iron and folic acid supplements during pregnancy had a significantly lowering incidence of LBW, in agreement with a study from Bangladesh [22]. This is further supported by a randomized, double-blind controlled trial comparing standard iron supplementation with multiple micronutrients during pregnancy [26]. Iron-alone supplementation could protect against low birthweight as compared to multiple micronutrients supplementation [26]. In addition, an overview of controlled trials suggested a 41% decline in the prevalence of intrauterine growth retardation with folic acid supplementation [27].

Furthermore, our study reviled that anaemic mothers had higher odds to deliver LBW neonates, consistent with a study in Yemen [12]. Micronutrient deficiency during pregnancy has been shown to have serious implications on the developing foetus and hence, birthweight. Severe anaemia could impair oxygen delivery to the foetus and thus interfere with normal intrauterine growth.

Consequences of inadequate nutritional intake and poor nutritional status not only directly affect women’s health status, but may also have a negative impact on birth- weight and early development. Our findings were consistent with a study from rural Oromia in Ethiopia, in which women in the inadequate MDD-W group had an increased risk of LBW and PTB [21]. Similarly, a study from Ghana showed that women dietary diversity scores and dietary patterns were found to be protective against low birthweight [13]. However, a recent randomized trial in India of increased consumption of dairy, fruits, and green leafy vegetables before and during pregnancy through a specially formulated snack had no effect on birthweight [32]. This discrepancy might be due to differences in study population, geographical location, and study design.

Main strength of this study was taking birthweight within 1 hour after birth. Measurement of some explanatory variables were, however, prone to recall bias. Finally, findings of this study can have a significant implication for prevention of low birthweight and emphasis should be given to nutrition education, nutrition assessment, supplementation of iron-folic acid and prevention of anaemia during pregnancy.

## Conclusion and recommendation

Several factors were found to be associated with low birthweight. Lack of iron and folic acid supplementation, absence of nutritional counselling during pregnancy, not taking snacks during pregnancy, MUAC less than 23, maternal anaemia and inadequate MDD-W were identified to be significant predictors of LBW. Governmental and non-governmental organizations working on maternal and child health should focus on identified factors in order to tackle the problem of LBW.

## Data Availability

All relevant data are within the paper and its Supporting Information.

## Abbreviations

AOR: adjusted odds ratio
CI: confidence interval
HFIAS: Household Food Insecurity Access Scale
LBW: Low Birthweight
MDD-W: Minimum dietary diversity score of women
MUAC: Mid upper arm circumference
VIF: Variable inflation factor
WHO: World health organization

## References

[1] UNICEF (2004). WHO low birth-weight: country, regional and global estimates.

[2] Strategies to promote optimal fetal growth and minimize the prevalence of LBW in Sri Lanka: health sector response: Family Health Bureau ministry of health; 2013.

[3] WHO (2014). WHO global nutrition targets 2025: low birth-weight policy brief.

[4] Ethiopian central statistical agency, Ethiopian demographic and health survey (EDHS), Ethiopian central statistical agency: Addis Abeba, Ethiopia, 2012.

[5] WHO guideline on optimal feeding of low birth-weight in low and middle income countries. Geneva, World health organization, 2011. 26042325

[6] Goldenberg RL, Culhane JF (2007). Low birth-weight in the United States. Am J Clin Nutr.

[7] Ramakrishnan U (2004). Nutrition and low birth-weight: from research to practice. Am J Clin Nutr.

[8] Barker DJP, Godfrey KM, Gibney MJ, Margetts BM, Kearney JM, Arab L (2004). Maternal nutrition, fetal programming and adult chronic disease. Public health nutrition.

[9] Torres-Arreola LP, Constantino-Casas P, Flores-Hernandez JPV-B, Rendon-Macias E (2005). Socioeconomic factors and low birth-weight in Mexico. BMC Public Health.

[10] Ghimire R, Phalke DB, Phalke VD, Banjade B, Singh AK (2014). Determinants of low birth-weight: a case control study in Pravara rural hospital in western Maharashtra. India IJSR.

[11] Khan A, Deeba F, Jaleel R (2016). Frequency and risk factors of low birth-weight in term pregnancy. PJMS.

[12] Muftah S (2016). Maternal under-nutrition and anaemia factors associated with low birth-weight babies in Yemen. Int J Community Med Public Health.

[13] Abubakari A, Jahn A (2016). Maternal dietary patterns and practices and birth-weight in northern Ghana. journalpone PLoS ONE.

[14] Shakya KL (2015). Key factors associated with low birth-weight at term in Nepal: a case control study. Int J Clin Biomed Res.

[15] Sharma SR (2015). Low birth-weight at term and its determinants in a tertiary hospital of Nepal: a case control study. PLoS One.

[16] Muchemi OM, Echoka E, Makokh A (2015). Factors associated with low birth-weight among neonates born at Olkalou district hospital, central region, Kenya. Pan Afr Med J.

[17] Sutan R, Mohtar M, Mahat AN, Tamil AM (2014). Determinant of low birth-weight infants: a matched case control study. OJPM.

[18] Kayode GA (2014). Contextual risk factors for low birth-weight: a multilevel analysis. PLoS One.

[19] Coates J, Swindale A, Bilinsky P (2007). Household food insecurity access scale (HFIAS) for measurement of household food access: Indicator guide (v.3).

[20] Megabiaw B, Zelalem M, Mohammed N (2012). Incidence and correlates of low birth weight at a referral hospital in Northwest Ethiopia. Pan Afr Med J.

[21] Tema T (2006). Prevalence and determinants of low birth-weight in Jimma zone, south West Ethiopia. EAMJ.

[22] Matin A et al. Maternal socioeconomic and nutritional determinants of low birth-weight, Shaheed Shohrawardy, medical college hospital, Dhaka, Bangladesh, J Dhaka Med Coll 2008; 17(2) : 83–87.

[23] Zerfu TA, Umeta M, Baye K (2015). Dietary diversity during pregnancy is associated with reduced risk of maternal anemia, preterm delivery, and low birth-weight in a prospective cohort study in rural Ethiopia. Am J Clin Nutr.

[24] Rizvi SA, Hatcher J, Jehan I, Qureshi R (2007). Maternal risk factors associated with low birth-weight in Karachi: a case control study. East Mediterr Health J.

[25] Mitchell EA, Robinson E, Clark PM, Becroft DMO, Glavish N, Pattison NS (2004). Maternal nutritional risk factors for small for gestational age babies in a developed country. Arch Dis Child Fetal Neonatal Ed.

[26] Ramakrishnan U, González T, Lynnette M, Rivera J, Martorell R (2003). Multiple micronutrient supplementation during pregnancy does not lead to greater infant birth size than does iron-only supplementation: a randomized controlled trial in a semirural community in Mexico. Am J Clin Nutr.

[27] Christian P, Keith P, Subarna K, Steven C, Elizabeth K (2003). Joanne K et.al. Effects of maternal micronutrient supplementation on fetal loss and infant mortality: a cluster-randomized trial in Nepal. Am J Clin Nutr.

[28] Dubey K, Nath C (2016). An epidemiological model investigating the association between mothers nutritional status and low birth-weight in India. Health.

[29] Muthayya S (2009). Maternal nutrition & low birth-weight - what is really important?. Indian J Med Res.

[30] Kim D, Saad A (2013). The social determinants of infant mortality and birth outcomes in western developed nations: across country systematic review. Int J Environ Res Public Health.

[31] Potdar D, Sahariah A, Gandhi M, Kehoe H, Brown N, Sane H (2014). Improving women’s diet quality preconceptionally and during gestation: effects on birth-weight and prevalence of low birth-weight-a randomized controlled efficacy trial in India (Mumbai maternal nutrition project). Am J Clin Nutr.

[32] Ahmed S, Hassen K, Wakayo T (2018). A health facility based case-control study on determinants of low birth weight in Dassie town, Northeast Ethiopia: the role of nutritional factors. BMC Nutrition Journal.

